# Synthesis of pentafluorosulfanyl (SF_5_) containing aromatic amino acids

**DOI:** 10.1016/j.jfluchem.2018.06.006

**Published:** 2018-08

**Authors:** Lucas Grigolato, William D.G. Brittain, Alex S. Hudson, Maria M. Czyzewska, Steven L. Cobb

**Affiliations:** Department of Chemistry, Durham University, South Road, Durham, DH1 3LE, United Kingdom

**Keywords:** Amino acid, Pentafluorosulfanyl, SF_5_, Negishi cross-coupling

## Abstract

Herein, a series of aromatic pentafluorosulfanyl (SF_5_) containing amino acids are reported. A Negishi cross-coupling strategy utilising a catalyst system of Pd(dba)_2_ and SPhos afforded the aforementioned SF_5_ amino acids in yields between 32% and 42%. Two dipeptides utilising both the amine and carboxylic functionalities of the synthesised SF_5_ containing amino acids were prepared, demonstrating their compatibility with common amide/peptide coupling reagents and strategies.

## Highlights

• First synthesis of a pentafluorosulfanyl containing aromatic amino acid.
• Amino acids were obtained through a Negishi cross-coupling strategy.
• SPhos ligand was found to deliver a superior yield of the amino acids.
• Two dipeptides with SF_5_ containing amino acids were prepared.
• The SF_5_ containing aromatic amino acids are compatible with commonly utilised peptide synthesis and deprotection strategies

## Graphical abstract

Herein, a series of aromatic pentafluorosulfanyl (SF_5_) containing amino acids are reported. A Negishi cross-coupling strategy utilising a catalyst system of Pd(dba)_2_ and SPhos afforded the aforementioned SF_5_ amino acids in yields between 35% and 42%. Two dipeptides utilising both the amine and carboxylic functionalities of the synthesised SF_5_ containing amino acids were prepared, demonstrating their compatibility with common amide/peptide coupling reagents and strategies.

**Figure:**
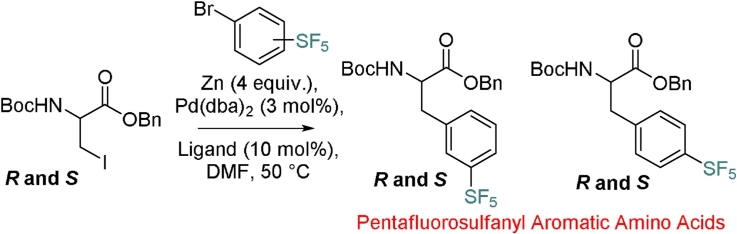

## 1 Introduction

The synthesis of novel amino acids is an area of considerable interest as it offers a route to access not only previously inaccessible natural products but also to modulate the properties of peptides [[1], [2], [3]]. Furthermore, the ability to include additional functionality (*e.g.* NMR probes, handles for chemical modification or bioconjugation) within peptide sequences has led to increasing interest in the synthesis of unusual amino acids in areas such as medicinal chemistry [4] and drug discovery [5,6].

Fluorine atoms have been demonstrated to modulate the structure, stability and activity of peptides [7]. For example, Meng and Kumar demonstrated that introduction of fluorine atoms into antimicrobial peptides increased their bacteriostatic activity or improved their stability towards protease degredation [8]. Therefore, the ability for peptide chemists to be able to readily access amino acid building blocks containing fluorine atoms is of the upmost importance.

The pentafluorosulfanyl (SF_5_) functional group is a moiety which at present is difficult to introduce into peptide sequences even though it has garnered attention across many research areas. The steric bulk and electronics of the SF_5_ group can modify both the conformation and chemical properties of a compound. This has led to the SF_5_ group being utilised to develop new pharmaceuticals [9] and agrochemicals [10].

The SF_5_ group has also been used as a substitute for the trifluoromethyl (CF_3_) group [11], however their physical and chemical properties differ quite considerably [12]. For instance, the geometry, the electron density profile, chemical/thermal stability and volume of the two groups are all very different. In addition due to their steric and electronic properties SF_5_ groups have also been investigated as replacements for *tert*-butyl [13], halogen [9] and nitro [14] functionalities.

**Scheme 1:**
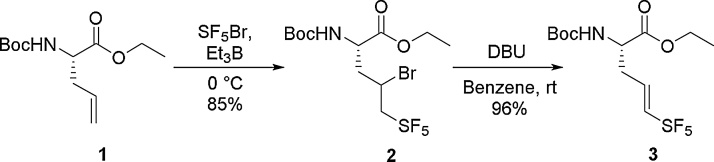
Previously accessed SF_5_ containing amino acid, Welch and co-workers [25].

There are a variety of ways to access alkyl [[15], [16], [17], [18]] and aryl [[19], [20], [21], [22], [23], [24]] pentafluorosulfanyl compounds. Despite the advances in the utility and synthesis of SF_5_ building blocks, the ability to introduce them into peptides has so far been limited [25]. To the best of our knowledge only one previous synthesis of an SF_5_ amino acid has been disclosed. Welch and co-workers reported a six step synthesis of an SF_5_ containing allyl glycine derivative **3** and further elaborated the amino acid into a heptapeptide (Scheme 1) [25].

In order to further explore the SF_5_ group in the field of peptide chemistry, we targeted the synthesis of pentafluorosulfanyl phenylalanine derivatives. Herein, we report the first synthesis of SF_5_ aromatic amino acids utilising a Negishi cross-coupling as a key C—C bond forming step.

## 2 Results and discussion

Negishi cross-coupling has been used to access a wide range of amino acids previously within the literature [26,27]. Therefore to begin with a halogenated amino acid precursor **4** was selected as one coupling partner for our Negishi strategy. The two enantiomers of the benzyl protected iodo cross-coupling partner were synthesised from the corresponding Boc protected serines as previously reported [28].

Next a Negishi cross-coupling reaction was carried out between iodo-alanine **4*S*** and commercially available 1-bromo-4-(pentafluorosulfanyl)benzene. Iodo-alanine **4*S*** was treated with 4 equivalents of zinc in dry DMF. The formed alkyl zinc species was reacted with 1-bromo-4-(pentafluorosulfanyl)benzene as the other coupling partner. The reaction was carried out with a tri(*o*-tolyl)phosphine (P(*o*-tol)_3_) ligand (10 mol%) and a Pd(dba)_2_ catalyst (3 mol%). This gave a disappointing 8% yield of the desired cross-coupling product **6*R*** (Table 1 Entry 1). When 1-bromo-3-(pentafluorosulfanyl)benzene was employed a yield of 26% of compound **5*R*** was obtained (Table 1 Entry 2). In an attempt to improve these yields the (P(*o*-tol)_3_) ligand was switched for SPhos. SPhos has been previously shown by Jackson and co-workers to improve the yields of Negishi cross-coupled products in the reaction between amido zinc species and aryl bromides [29].

**Table 1:** Reaction optimisation of the key Negishi cross-coupling reaction.


Starting material	Ligand	Bromo-phenyl-SF_5_	Product	Yield (%)^a^
	P(*o*-tol)_3_		6*R*	8^b^
	P(*o*-tol)_3_		5*R*	26^b^
	SPhos		6*R*	38^b^
	SPhos		5*R*	35^b^
	SPhos		6*S*	42^c^
	SPhos		5*S*	32^c^

a) Refers to the isolated yield following flash column chromatography.

b) Reactions stirred at 50 °C for 5 h.

c) Reactions stirred at 50 °C for 3 h.

The addition of SPhos showed a marked improvement in the yields of the cross-coupled products. The yield of **6*R*** was increased to 38% (Table 1 Entry 3) and **5*R*** was similarly increased to 35% (Table 1 Entry 4). The opposite enantiomer, **4*R*** was also exposed to the SPhos/Pd(dba)_2_ mediated conditions garnering the SF_5_ containing species **6*S*** and **5*S*** in 42% and 32% yield respectively (Table 1 Entries 5 and 6).

**Fig. 1:**
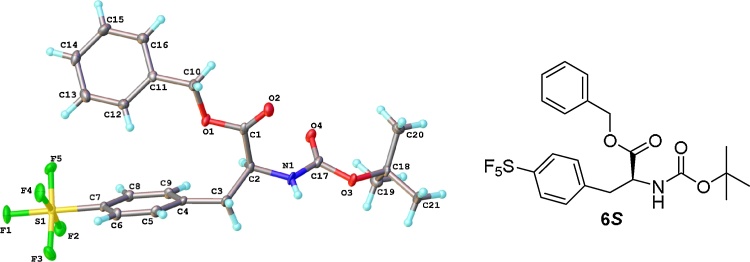
Crystal structure of compound 6*S.*

Compound **6*S*** was successfully crystallised through vapour diffusion of water and ethanol and a crystal structure was obtained (Fig. 1) [30]. The structure obtained for **6*S*** displayed an absolute configuration of *S* confirming our stereochemical assignment.

In order to demonstrate the compatibility of **5** and **6** with amide bond formation and in turn peptide synthesis we sought to form dipeptides with both of these synthesised pentafluorosulfanyl amino acids. In addition, we hoped that our choice of orthogonal protecting groups would mean that selective deprotection could be carried out without degradation of the amino acids occurring.

**Scheme 2:**
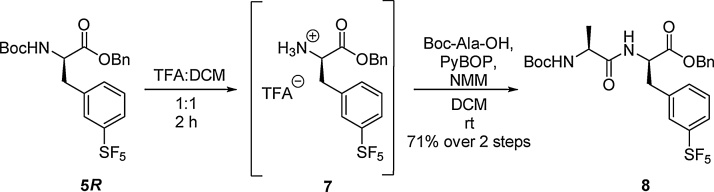
Formation of a dipeptide using SF_5_ amino acid 5*R.*

Boc deprotection of **5*R*** was carried out using standard TFA mediated reaction conditions. The deprotected amino acid was reacted directly with Boc—Ala—OH in the presence of PyBOP and *N*-methyl morpholine (NMM). The amide bond forming reaction proceeded smoothly to garner the desired dipeptide **8** in a 71% yield, over the two steps (Scheme 2).

**Scheme 3:**
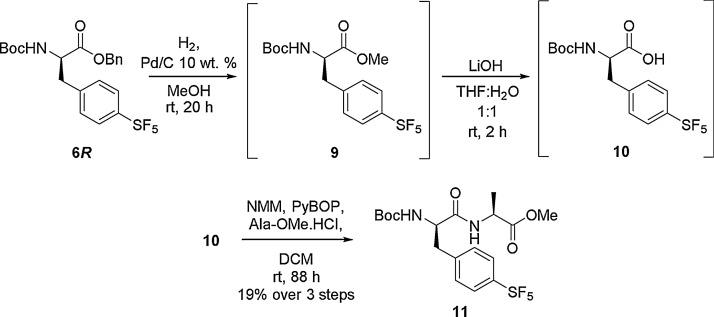
Formation of a dipeptide using SF_5_ amino acid 8*R.*

Next the benzyl protected amino acid **6*R*** was deprotected through hydrogenation with palladium on charcoal to give the corresponding methyl ester **9**, which was subsequently deprotected to the free carboxylic acid **10** using lithium hydroxide. This material was directly coupled with Boc—Ala—OH in the presence of PyBOP and NMM. This reaction afforded the dipeptide **11** in a 19% yield over 3 steps (Scheme 3).

## 3 Conclusion

We have demonstrated that SF_5_ containing aromatic amino acids can be accessed through the Negishi cross-coupling of pentafluorosulfanyl aryl bromides with amido alkyl iodide species. The use of SPhos as a ligand for palladium helped to increase the yields for the cross-couplings. This methodology is to the best of our knowledge only the second disclosed route to an SF_5_ containing amino acid and the first to yield an aromatic SF_5_ amino acid. The synthesised aryl-SF_5_ amino acids were shown to be compatible with standard amide/peptide bond coupling reagents and de-protection strategies. We are now exploring the application of the newly formed aromatic SF_5_ amino acids in larger peptide sequences, as a means to modify their chemical and physical properties.

## 4 Experimental

### 4.1 General

All starting materials and reagents were bought from commercial sources and used as received. ^1^H NMR spectra were recorded at 400, 600 and 700 MHz using Bruker Avance III, Varian VNMR(S)-600 and Varian VNMR(S)-700 spectrometers. ^13^C NMR spectra were recorded at 100, 151 and 176 MHz using Bruker Avance III, Varian VNMR(S)-600 and Varian VNMR(S)-700 spectrometers. ^19^F NMR spectra were recorded at 376 MHz using a Bruker Avance III spectrometer. All coupling constants are reported in Hertz (Hz). In cases where it was required 2D NMR techniques were used to confirm compound identity. Chemical shifts are reported in ppm and are referenced to residual solvent peaks; CHCl_3_ (^1^H 7.26 ppm, ^13^C 77.0 ppm) and DMSO (^1^H 2.50 ppm, ^13^C 39.5 ppm). Mass spectra were collected on a Waters TQD mass spectrometer and accurate mass spectra were collected on a Waters LCT Premier XE mass spectrometer. Optical rotations were measured with a Jasco P-1020 polarimeter at room temperature.

### 4.2 General method for Negishi cross-coupling

Procedure A:

Zinc dust (0.193 g, 2.95 mmol, 4.0 equiv.) was heated at 100 °C under vacuum for 30 min A catalytic amount of iodine in dry DMF (0.5 mL) was added and heated at 70 °C under argon for 20 min. Iodo-alanine derivative **4** (0.300 g, 0.74 mmol, 1.0 equiv.) in dry DMF (0.5 mL) was added and the reaction mixture heated at 50 °C for 20 min. After this time the corresponding (Bromophenyl)sulfur pentafluoride (0.209 g, 0.74 mmol, 1.0 equiv.), Pd(dba)_2_ (0.012 g, 3 mol%) and P(*o*-tol)_3_ (0.022 g, 0.073 mmol, 0.1 equiv.) were added to the reaction mixture and the reaction mixture stirred at 50 °C for 5 h followed by 24 h at rt. The crude material was purified directly by flash column chromatography (SiO_2_ 100:0 to 0:100 hexane:DCM).

Procedure B:

The reaction was carried out in the same manner as described in procedure A with P(*o*-tol)_3_ replaced by SPhos (0.030 g, 0.074 mmol, 0.1 equiv.).

### 4.3 Synthesis of benzyl (*R*)-2-((tert-butoxycarbonyl)amino)-3-(3-(pentafluoro-l6-sulfanyl)phenyl)propanoate 5*R*

Compound **5*R*** was synthesised according to the general Negishi cross-coupling procedures

Procedure A: 0.091 g, 26%

Procedure B: 0.122 g, 35%

^1^H NMR (400 MHz, CDCl_3_) δ 7.61 (d, *J* = 8.5, 1H, Ar*H*), 7.49 (s, 1H, Ar*H*), 7.39-7.35 (m, 3H, Ar*H*), 7.32-7.27 (m, 3H, Ar*H*), 7.15 (d, *J* = 7.3, 1H, Ar*H*), 5.14 (s, 2H, OC*H_2_*), 5.04 (d, *J* = 7.1, 1H, N*H*), 4.65 (app. q, *J* = 5.8, 1H, *H*_α_), 3.22 (dd, *J* = 13.8, 5.8, 1H, *H*_β_), 3.10 (dd, *J* = 13.8, 5.8, 1H, *H*_β_), 1.41 (s, 9H, Boc-C*H_3_*); ^13^C NMR (101 MHz, CDCl_3_) δ 171.2, 155.0, 154.1, 154.1, 154.0, 137.4, 135.0, 132.7, 128.9, 128.83, 128.81, 128.7, 127.1, 124.8, 124.7, 124.7, 80.4, 67.6, 54.3, 38.3, 28.4; ^19^F -NMR (376 MHz, CDCl_3_) 84.56 (quint., *J* = 150.4, 1 F), 62.83 (d, *J* = 150.4, 4 F). HRMS ESI^+^ Calculated for [M+H]^+^ C_21_H_25_F_5_NO_4_S^+^ = 482.1424 Found = 482.1443; ${\left[\alpha \right]}_{D}^{27}$ = −18.76 (c = 1, CH_2_Cl_2_)

### 4.4 Synthesis of benzyl (*R*)-2-((tert-butoxycarbonyl)amino)-3-(4-(pentafluoro-l6-sulfanyl)phenyl)propanoate 6*R*

Compound **6*R*** was synthesised according to the general Negishi cross-coupling procedure

Procedure A: 0.030 g, 8%

Procedure B: 0.135 g, 38%

^1^H NMR (400 MHz, CDCl_3_) δ 7.57 (d, *J* = 8.3, 2H, Ar*H*), 7.43 – 7.34 (m, 3H, Ar*H*), 7.30 – 7.26 (m, 2H, Ar*H*), 7.10 (d, *J* = 8.1, 2H, Ar*H*), 5.19 (d, *J* = 12.1, 1H, OC*H_2_*), 5.09 (d, *J* = 12.1, 1H, OC*H_2_*), 5.03 (d, *J* = 8.1, 1H, N*H*), 4.65 (app. q, *J* = 6.7, 1H, *H*_α_), 3.17 (dd, *J* = 13.8, 6.0, 1H, *H*_β_), 3.07 (dd, *J* = 13.8, 6.0, 1H, β-*H*_β_), 1.41 (s, 9H, Boc-C*H_3_*); ^13^C NMR (101 MHz, CDCl_3_) δ 171.3, 155.0, 152.8, 140.2, 135.0, 129.8, 128.9, 128.8, 126.2, 126.1, 126.1, 80.4, 67.6, 54.2, 38.1, 28.4; ^19^F NMR (376 MHz, CDCl_3_) δ 84.74 (quint., *J* = 150.4, 1 F), 63.01 (d, *J* = 150.4, 4 F). HRMS ESI^+^ Calculated for [M+H]^+^ C_21_H_25_F_5_NO_4_S^+^ = 482.1424 Found = 482.1433; ${\left[\alpha \right]}_{D}^{27}$ = −10.36 (c = 1, CH_2_Cl_2_).

### 4.5 Synthesis of benzyl (*S*)-2-((tert-butoxycarbonyl)amino)-3-(3-(pentafluoro-l6-sulfanyl)phenyl)propanoate 5*S*

Compound **5*S*** was synthesised according to the general Negishi cross-coupling procedure B with the following modification. The reaction was stirred for 3 h at 50 °C followed by 24 h at rt to give the product in a 32% (0.225 g) yield.

^1^H NMR (400 MHz, CDCl_3_) δ 7.61 (dd, *J* = 8.2, 2.2, 1H, Ar*H*), 7.50 (s, 1H, Ar*H*), 7.40-7.35 (m, 3H, Ar*H*), 7.34-7.28 (m, 3H, Ar*H*), 7.16 (d, *J* = 7.6, 1H, Ar*H*), 5.14 (s, 2H, OC*H_2_*), 5.10 (d, *J* = 8.2, 1H, N*H*), 4.66 (app. q, *J* = 6.5, 1H, *H*_α_) 3.23 (dd, *J* = 13.9, 6.0, 1H, *H*_β_), 3.11 (dd, *J* = 13.9, 6.0, 1H, *H*_β_), 1.42 (s, 9H, Boc-C*H_3_*); ^13^C NMR (101 MHz, CDCl_3_) δ 171.2, 155.0, 154.2, 154.1, 153.9, 137.4, 135.0, 132.6, 128.9, 128.8, 128.8, 128.7, 127.1, 124.7, 124.7, 124.7, 80.3, 67.5, 54.3, 38.2, 28.3; ^19^F NMR (376 MHz, CDCl_3_) δ 84.60 (quint., *J* = 149.8, 1 F), 62.84 (d, *J* = 149.8, 4 F); HRMS ESI^+^ Calculated for [M+H]^+^ C_21_H_25_F_5_NO_4_S^+^ = 482.1424 Found = 482.1436; $\;{\left[\alpha \right]}_{D}^{27}$ = +19.08 (c = 1, CH_2_Cl_2_).

### 4.6 Synthesis of benzyl (*S*)-2-((tert-butoxycarbonyl)amino)-3-(4-(pentafluoro-l6-sulfanyl)phenyl)propanoate 6*S*

Compound **6*S*** was synthesised according to the general Negishi cross-coupling procedure B with the following modification. The reaction was stirred for 3 h at 50 °C followed by 24 h at rt to give the product in a 42% (0.300 g) yield.

^1^H NMR (400 MHz, CDCl_3_) δ 7.57 (d, *J* = 8.3, 2H, Ar*H*), 7.39 – 7.34 (m, 3H, Ar*H*), 7.30 – 7.26 (m, 2H, Ar*H*), 7.10 (d, *J* = 8.2, 2H, Ar*H*), 5.19 (d, *J* = 12.1, 1H, OC*H_2_*), 5.09 (d, *J* = 12.1, 1H, OC*H_2_*), 5.03 (d, *J* = 8.1, 1H, N*H*), 4.65 (m, 1H, *H*_α_), 3.17 (dd, *J* = 13.8, 6.0, 1H, *H*_β_), 3.07 (dd, *J* = 13.8, 6.0, 1H, *H*_β_), 1.41 (s, 9H, Boc-C*H_3_*); ^13^C NMR (101 MHz, CDCl_3_) δ 171.3, 155.0, 152.8, 140.2, 135.0, 129.8, 128.9, 128.8, 126.2, 126.1, 126.1, 80.4, 67.6, 54.2, 38.1, 28.4; ^19^F NMR (376 MHz, CDCl_3_) δ 84.58 (quin., *J*  = 156.8 Hz, 1 F), 62.97 (d, *J* = 156.8 Hz, 4 F); HRMS ESI^+^ Calculated for [M+H]^+^ C_21_H_25_F_5_NO_4_S^+^ = 482.1424 Found = 482.1446; $\;{\left[\alpha \right]}_{D}^{27}$ = +10.78 (c = 1, CH_2_Cl_2_).

*Crystal data for* **6*S***: C_21_H_24_F_5_NO_4_S, M = 481.47, monoclinic, space group P 2_1_, a = 13.8424(11), b = 5.4800(5), c = 15.2791(12) Å, β = 113.218(3)°, U = 1065.15(15) Å^3^, F(000) = 500.0, Z = 2, D_c_ = 1.501 mg m^−3^, μ = 0.224 mm^-1^ (Mo-Kα, λ = 0.71073 Å), T = 120(1)K. 21,584 reflections were collected on a Bruker D8Venture (Photon100 CMOS detector, Iμ(S)-microsource, focusing mirrors, shutterless mode, 1° ω-scan) diffractometer yielding 5393 unique data (R_merg_ = 0.0614). The structure was solved by direct method and refined by full-matrix least squares on F^2^ for all data using SHELXTL and OLEX2 software [31,32]. All non-hydrogen atoms were refined with anisotropic displacement parameters; H-atoms were placed into calculated positions and refined in riding mode. Final wR_2_(F^2^) = 0.1651 for all data (385 refined parameters), conventional R_1_(F) = 0.066 for 4374 reflections with I ≥ 2σ, GOF = 1.075. The absolute configuration of the compound has been established by measurements of anomalous dispersion effects (Flack parameter (x) = 0.1(1), Hooft parameter (y) = 0.07(4)). Crystallographic data for the structure have been deposited with the Cambridge Crystallographic Data Centre as supplementary publication CCDC-1837505.

### 4.7 Synthesis of benzyl (*R*)-2-((*S*)-2-((tert-butoxycarbonyl)amino)propanamido)-3-(3-(pentafluoro-l6-sulfanyl)phenyl)propanoate 8

To a stirred solution of Boc-D-(3-SF_5_)-Phe-OBn (**5*R***) (0.110 g, 0.23 mmol) in DCM (4 mL) was added TFA (4 mL) and the resulting solution was stirred at room temperature for 2 h. The reaction mixture was concentrated under reduced pressure and any residual TFA was removed by co-evaporation with ether. The recovered material was suspended in DCM (2 mL) and NMM (0.087 g, 0.86 mmol, 0.08 mL) added, the resulting solution was stirred for 5 min at rt. PyBOP (0.120 g, 0.23 mmol) and Boc—Ala—OH (0.044 g, 0.23 mmol) dissolved in DCM (2 mL) were then added and the reaction mixture left to stir for 15 h at rt. The reaction mixture was concentrated under reduced pressure and purified *via* column chromatography (100:0 to 0:100, hexane/EtOAc). Dipeptide **8** was afforded as a cream solid in a 71% (0.090 g) yield over 2 steps.

^1^H NMR (400 MHz, CDCl_3_) δ 7.61 (ddd, *J* = 8.3, 2.3, 1.0, 1H, Ar*H*), 7.47 (t, *J* = 1.9, 1H, Ar*H*), 7.40 – 7.34 (m, 3H, ArH), 7.34 – 7.27 (m, 3H, Ar*H*), 7.18 (d, *J* = 7.6, 1H, Ar*H*), 6.72 (s, 1H, N*H*), 5.13 (app s, 2H, Bn-C*H_2_*), 4.90 (app. q, *J* = 6.1, 1H, Phe (3-SF_5_)-*H_α_*), 4.84 (bs, 1H, N*H*), 4.16 (m, 1H, Ala-*H_α_*), 3.23 (dd, *J* = 14.0, 5.8, 1H, Phe (3-SF_5_)-*H_β_*), 3.16 (dd, *J* = 14.0, 5.8, 1H Phe (3-SF_5_)-*H_β_*), 1.41 (s, 9H, Boc-C*H_3_*), 1.30 (d, *J* = 7.1, 3H, Ala- *H_β_*); ^13^C NMR (101 MHz, CDCl_3_) δ 172.6, 170.8, 155.6, 154.3, 154.1, 154.0, 137.2, 134.9, 132.7, 129.0, 128.8, 128.6, 126.9, 126.9, 126.8, 124.8, 124.8, 124.7, 80.4, 67.7, 53.2, 50.1, 37.8, 29.8, 28.4, 18.2; ^19^F NMR (376 MHz, CDCl_3_) δ 84.46 (quin., *J* = 149.9, 1 F), 62.79 (d, *J* = 149.9, 4 F); HRMS ESI^+^ Calculated for [M+H]^+^ C_24_H_30_F_5_N_2_O_5_S^+^ = 553.1796 Found = 553.1797.

### 4.8 Synthesis of methyl ((*R*)-2-((tert-butoxycarbonyl)amino)-3-(4-(pentafluoro-l6-sulfanyl)phenyl)propanoyl)-L-alaninate 11

To a mixture of Boc-D-(4-SF_5_)-Phe-OBn (**6*R***) (0.075 g, 0.16 mmol) and Pd/C 10 wt% under an inert atmosphere was added dry methanol (10 mL). The solution was stirred for 24 h under a positive pressure of hydrogen. The solution was concentrated under reduced pressure and the residue taken up in THF (1 mL). The resulting solution was cooled to 0 °C before addition of a solution of LiOH (0.014 g, 2 equiv.) in water (0.5 mL), the reaction was stirred for 2 h at 0 °C. The reaction mixture was then concentrated under reduced pressure. The remaining solution was acidified to pH ≈ 3 with an aqueous solution of citric acid (10%w/v). The solution was extracted with EtOAc (3 x 5 mL). The combined organic layers were washed with brine (5 mL), dried over MgSO_4_ and concentrated under reduced pressure. The recovered oil was dissolved in DCM (2 mL) and PyBOP (0.084 g, 1 equiv.) added, the resulting solution was stirred at room temperature. In a separate flask, NH_2_-Ala-OMe.HCl (0.023 g, 1 equiv.) was dissolved in DCM (2 mL), NMM (0.06 mL, 3 equiv.) added and the resulting solution stirred for 5 min. The two solutions were mixed together and stirred at room temperature for 86 h. The reaction mixture was concentrated under reduced pressure and the residue purified *via* column chromatography (100% hexane to 100% EtOAc) to afford dipeptide **11** as a cream solid in a 19% (0.015 g) yield over 3 steps.

^1^H NMR (400 MHz, CDCl_3_) δ 7.68 (d, *J* = 8.4, 2H, Ar*H*), 7.31 (d, *J* = 8.4, 2H, Ar*H*), 6.45 (d, *J* = 7.4, 1H, NH), 4.95 (br s, 1H, N*H*), 4.52 (app t, *J* = 7.2, 1H, Ala-*H_α_*), 4.41 (br s, 1H, SF_5_-Phe-*H*_α_), 3.74 (s, 3H, OC*H_3_*), 3.19 (dd, *J* = 14.0, 7.0, 1H, SF_5_-Phe-*H_β_*), 3.05 (dd, *J* = 14.0, 7.0, 1H, SF_5_-Phe-*H_β_*), 1.39 (s, 9H, C*H_3_*), 1.31 (d, *J* = 7.1, 3H, Ala-H_β_); ^13^C NMR (176 MHz, CDCl_3_) δ 172.89, 170.00, 140.81, 129.67, 126.15, 55.21, 52.53, 48.02, 37.83, 29.67, 28.16, 18.11. ^19^F NMR (376 MHz, CDCl_3_) δ 84.58 (quin., *J* = 156.8, 1 F), 62.97 (d, *J* = 156.8, 4 F). HRMS ESI^+^ Calculated for [M+H]^+^ C_18_H_35_F_5_N_2_O_5_S^+^ = 477.1490 Found = 477.1483.
